# GUIP1: a R package for dose escalation strategies in phase I cancer clinical trials

**DOI:** 10.1186/s12911-020-01149-3

**Published:** 2020-06-24

**Authors:** D. Dinart, J. Fraisse, D. Tosi, A. Mauguen, C. Touraine, S. Gourgou, M. C. Le Deley, C. Bellera, C. Mollevi

**Affiliations:** 1 Inserm CIC1401, Module Epidémiologie Clinique, Institut Bergonié, Bordeaux, France; 2 Institut du Cancer Montpellier (ICM), Université de Montpellier, Montpellier, France; 3 Memorial Sloan Kettering Cancer Center, New York, USA; 4 Centre Oscar Lambret, Lille, France; 5 Université Paris-Saclay, Université Paris-Sud, UVSQ, CESP, INSERM, Villejuif, France; 6 Institut de Recherche en Cancérologie de Montpellier INSERM U1194, Université de Montpellier, Montpellier, France

**Keywords:** Phase 1 clinical trial, Graphical user interface, Dose escalation design

## Abstract

**Background:**

The main objective of phase I cancer clinical trials is to identify the maximum tolerated dose, usually defined as the highest dose associated with an acceptable level of severe toxicity during the first cycle of treatment. Several dose-escalation designs based on mathematical modeling of the dose-toxicity relationship have been developed. The main ones are: the continual reassessment method (CRM), the escalation with overdose control (EWOC) method and, for late-onset and cumulative toxicities, the time-to-event continual reassessment method (TITE-CRM) and the time-to-event escalation with overdose control (TITE-EWOC) methods. The objective of this work was to perform a user-friendly R package that combines the latter model-guided adaptive designs.

**Results:**

GUIP1 is an R Graphical User Interface for dose escalation strategies in Phase 1 cancer clinical trials. It implements the CRM (based on Bayesian or maximum likelihood estimation), EWOC and TITE-CRM methods using the dfcrm and bcrm R packages, while the TITE-EWOC method has been specifically developed. The program is built using the TCL/TK programming language, which can be compiled via R software libraries (tcltk, tkrplot, tcltk2). GUIP1 offers the possibility of simulating and/or conducting and managing phase I clinical trials in real-time using file management options with automatic backup of study and/or simulation results.

**Conclusions:**

GUIP1 is implemented using the software R, which is widely used by statisticians in oncology. This package simplifies the use of the main model-based dose escalation methods and is designed to be fairly simple for beginners in R. Furthermore, it offers multiple possibilities such as a full traceability of the study. By including multiple innovative adaptive methods in a free and user-friendly program, we hope that GUIP1 will promote and facilitate their use in designing future phase I cancer clinical trials.

## Background

The development of cancer drugs in a clinical setting requires a well-codified procedure. Phase I studies of a new treatment are usually the first to involve human subjects, and their aim is to select doses according to acceptable toxicity. The drug efficacy is then preliminarily tested in a phase II trial. Finally, a phase III trial compares the safety and efficacy of the new treatment against the current standard treatment prior to its approval for general use.

First-in-human (FIH) phase I studies of cancer drugs are performed on patients for whom no other therapeutic option is available. The main objective of phase I cancer clinical trials is to identify the maximum tolerated dose (MTD) of an experimental drug. Commonly, the MTD is associated with a predefined probability of unacceptable toxicity (20–33%), called the dose-limiting toxicity (DLT), which is evaluated in general during the first cycle of treatment. The recommended phase II dose (RP2D) is then suggested for subsequent phase II trials. Most of the available statistical methods used to design phase I clinical trials in oncology have been developed for cytotoxic conventional agents.

Two types of dose-escalation designs have been proposed: algorithm-based designs, such as the standard 3 + 3 design, and model-based designs. The 3 + 3 design is the simplest to interpret and implement [1], and it is the most commonly used phase I dose-escalation method. This method defines the MTD as the highest dose at which less than one-third of the treated patients experience intolerable toxicity during the first cycle of treatment. However, the 3 + 3 method underuses available trial data and is based only on empirical evidence without including statistical considerations; thus, this method allocates more patients to sub-therapeutic doses and presents lower operating characteristics than model-based methods [2, 3].

Model-based methods, being more computationally intensive and complex, are still underused in practice, although their use has gradually increased in recent years [4]. A barrier to their use is the need for software that requires specific skills. They are based on mathematical modeling of the dose-toxicity relationship. Specifically, the dose-toxicity curve is estimated using a parametric mathematical model that determines the probability of a DLT for the next patient to be included in the study, based on the doses and responses obtained for all previous patients. The continual reassessment method with either Bayesian estimation (CRMB) [5] or maximum likelihood estimation (CRML) [6] were the first two adaptive designs proposed. The CRMB and CRML models only incorporate complete observations, leading to a staggered accrual. In certain frameworks, such as the assessment of late-onset toxicities in radiotherapy-based trial, the observation period, and thus the trial duration, increases. Indeed, if one wants to wait for the full assessment of all previous observations period, then it is necessary to temporarily stop accrual, even though eligible patients who have been informed about the trial are immediately available. An extension of the CRM method, called time-to-event (TITE)-CRM [7], takes into account incomplete observations in a sequential re-estimation of the initial CRM model. Specifically, the likelihood is weighted by a function of the actual observation time of the patients and the total observation period defined for the study. For example, if a linear function is chosen, assuming 2 months of follow-up out of 4, the weight would be equal to 0.5.

The escalation with overdose control (EWOC) method [8] is the first dose-finding procedure that directly incorporates an ethical constraint to limit the probability of treating patients with excessively high doses. This constraint is applied to the CRM model via a parameter α, which represents the expected proportion of patients treated with doses higher than the MTD. The EWOC method approaches the MTD as quickly as possible while keeping the expected proportion of overdosed patients below the α value.

Note that others robust versions of the CRM method were also developed such Bayesian model averaging CRM [9] which proposes to model several parallel CRM with different prespecified toxicity probabilities or Bayesian data augmentation CRM [10] which addresses the late-onset toxicity problem. At last, CRM was extended to take into account ordinal toxicity outcomes [11, 12] or two competing outcomes [13].

More recently, a new class of models, the model-assisted designs, combining the simplicity of the algorithm-based designs and the performance of model based designs was introduced [14–16] such the modified toxicity probability interval (mTPI) design [17] or Bayesian optimal interval (BOIN) design [18] for example. Zhou et al. [15] showed that model-based designs, such as CRM, and model-assisted designs, such as BOIN, had similar performances and that we could favor one or the other depending on prior knowledge of the true dose-toxicity curve. However, despite good operating characteristics model-assisted designs are still underutilized.

Several software programs that implement dose-escalation designs, such as np1 [19], R packages: dfcrm, bcrm, and CRM ( [20], SAS macro TITE-CRM (https://sph.umich.edu/ccb/tite-resources.html), and biostatistics software proposed by MD Anderson: https://biostatistics.mdanderson.org/SoftwareDownload/ and the website named Trial Designs (https://www.trialdesign.org/), are available to conduct a phase I trial. Here, we propose a free and user-friendly R graphical interface, named GUIP1, that combines different model-guided adaptive designs (CRMB, CRML, EWOC, TITE-CRM, TITE-EWOC) for simulating and conducting phase I cancer clinical trials. This software aims to facilitate the design and analysis of phase I trials by offering an easy-to-use interface for entering the trial’s characteristics, a simulation setting to assess each model’s performance for the given trial, and a graphical output of the results. We used published R packages (dfcrm and bcrm) and implemented the TITE-EWOC method.

## Implementation

Model-based dose escalation designs in phase I clinical trials are generally based on the following two stages (for additional details, see [5–8, 21]).

First, clinical inputs are required, such as the number of dose levels and the first dose level to be investigated. Similarly, the DLT must be carefully defined, including the observation period. Based on previous publications on treatments used in similar clinical settings, the toxicity target level and the prior toxicity probabilities must be established for each dose level. Finally, a stopping rule needs to be chosen.

Second, the dose-toxicity relationship is modeled with the underlying assumption that the probability of DLT monotonically increases with dose levels.

### Definitions and notations

Let *n* be the number of patients included in the study. *Y*_1_, *Y*_2,_…, *Y*_*n*_ are the binary random variables defined for any patient *j* ∈ [1 : *n*] such that *Y*_*j*_ = 1 if patient j has DLT and *Y*_*j*_ = 0 otherwise. Let *X* be the dose variable. We consider the space *V* of the *k* dose levels administered: *V* = {*x*_1_, ≤*x*_2_ ≤ …*x*_*k*_}, where *x*_*i*_ is the dose associated with the *i*^*th*^ level. Let *Ω*_*j*_ = {(*x*_1_, *y*_1_), (*x*_2_, *y*_2_)…. (*x*_*j* − 1_, *y*_*j* − 1_)} be the history of the *j* − 1 first patients’ toxic responses and *θ* the target toxicity level.

### Continual reassessment method (CRM)

This method models the probability of toxic response for the *j*^*th*^ patient conditionally to the dose *x*_*i*_ by *ψ*(*x*_*i*_, *a*) = *P*(*Y*_*j*_ = 1| *X* = *x*_*i*_), where *i* ∈ [1. . *k*], *j* ∈ [1. . *n*], and *a* is the model parameter. It is assumed that there is an *a*_0_ > 0 such that *ψ*(*x*^⋆^, *a*_0_) = *θ* where *x*^⋆^ is the dose with the target toxicity level. In practice, once *a*_0_ obtained, the administered dose corresponds to the dose *x*_0_ ∈ *V*, such that *ψ*(*x*_0_, *a*_0_) is the closest to the targeted toxicity level *θ*. The CRM was initially developed in a Bayesian setting, and subsequently a frequentist approach was proposed [6]. The empiric power model $\left(\psi \left({x}_0,{a}_0\right)={x_0}^{\exp \left({a}_0\right)}\right)$ presented in the original paper is one option of modeling, but it is not the only option. Logistic regression modeling can also be used (one-parameter logistic or two-parameter logistic) with similar operating characteristics, especially when one parameter is fixed and only one parameter is estimated [22].

### Bayesian approach (CRMB)

Before the first inclusion (*j* = 1), a prior distribution is chosen for *a*_0_ which is denoted by *f*(*a*, *Ω*_1_) (for example, *f*(*a*, *Ω*_1_) =  *exp* (−*a*)). At the *j*^*th*^ patient enrollment (*j* > 1), the distribution of the parameter *a*_0_ is reassessed using Bayes’ theorem. We obtain the updated posterior *f*(*a*, *Ω*_*j*_) from

$$ f\left(a,{\varOmega}_j\right)=\frac{f\left(a,{\varOmega}_{j-1}\right)\times \prod \limits_{l=1}^{j-1}{\left[\psi \left({x}_l,a\right)\right]}^{y_l}{\left[1-\psi \left({x}_l,a\right)\right]}^{1-{y}_l}}{\int_0^{\infty}\prod \limits_{l=1}^{j-1}{\left[\psi \left({x}_l,u\right)\right]}^{y_l}{\left[1-\psi \left({x}_l,u\right)\right]}^{1-{y}_l} du} $$

where

1 $$ \prod \limits_{l=1}^{j-1}{\left[\psi \left({x}_l,a\right)\right]}^{y_l}{\left[1-\psi \left({x}_l,a\right)\right]}^{1-{y}_l} $$

(1) is the current likelihood of the model.

An updated estimate of parameter *a*_0_ is given by the conditional expectation of this distribution:

$$ {\hat{a}}_j=E\left[a|{\varOmega}_j\right]={\int}_0^{\infty } af\left(a,{\varOmega}_j\right) da $$

Then, the probability of DLT for the *j*^*th*^ patient at dose *x*_*i*_ is estimated by $\psi \left({x}_i,\hat{a}\right)={\int}_0^{\infty}\psi \left({x}_i,a\right)\times f\left(a,{\varOmega}_j\ \right) da$.

### Frequentist approach (CRML)

The aim of the frequentist approach is to address the need for estimating the Bayesian prior information. The parameter *a* of the distribution is estimated by maximizing the likelihood provided in Eq. (1) above. In contrast to the Bayesian approach, the maximum likelihood method requires an additional condition to get started. This is needed because the likelihood equation has no solution until at least one DLT and one non-DLT have been observed. Therefore, this method generally starts with an algorithm-based approach (for example, 3 + 3) until the first DLT is observed. The choice of a Bayesian approach versus the frequentist approach has a very little impact on the operating characteristics of the method [22].

### Time-to-event CRM (TITE-CRM)

In both TITE approaches, we choose to use a linear weight. Let *T* be the planned observation period, and we suppose that patient *j* was followed until *u*_*j*_ ≤ *T*. We define the weighting factor *w*_*j*_ for this patient by

$$ {w}_j=\left\{\begin{array}{c}\frac{u_j}{T}\kern0.5em \mathrm{if}\ \mathrm{patient}\ \mathrm{j}\ \mathrm{is}\ \mathrm{still}\ \mathrm{on}\ \mathrm{follow}-\mathrm{up}\ \mathrm{without}\ \mathrm{DLT}\kern10.50em \\ {}1\mathrm{if}\ \mathrm{patient}\ \mathrm{j}\ \mathrm{has}\ \mathrm{a}\ \mathrm{DLT}\kern22.50em \end{array}\right.\kern0.5em $$

The weighted likelihood of the model would thus simply be written as $\prod \limits_{l=1}^{j-1}{\left[{w}_l\times \psi \left({x}_l,a\right)\right]}^{y_l}{\left[1-{w}_l\times \psi \left({x}_l,a\right)\right]}^{1-{y}_l}$. The weights are now part of the likelihood of the model, which affect the $\hat{a}$-parameter posterior distribution. Note that the TITE-CRM method is only proposed in a Bayesian setting in GUIP1.

### Escalation with overdose control (EWOC)

EWOC is a Bayesian dose-finding design that produces a consistent sequence of doses while controlling for the probability of overdose. This ethical constraint is the main difference between the EWOC and CRM methods. In other words, EWOC ensures that the expected proportion of patients receiving a dose greater than the MTD (overdose) does not exceed a pre-specified value α, *the feasibility bound*. The EWOC method selects, at each new inclusion j, the dose level such that the conditional probability of exceeding the MTD, defined by *π*_*j*_(*x*) = *P*(*MTD* ≤ *x* | *Ω*_*j*_), is inferior or equal to the parameter α.

A two-parameter logistic model was initially considered to model the dose-toxicity relationship [8]. By denoting *a* = (*a*_0_, *a*_1_) as the unknown parameter vector, the probability of toxic response for the *j*^*th*^ patient at dose x_i_ is estimated to be $\psi \left({x}_{i,}a\right)=\frac{\mathit{\exp}\left({a}_0+{a}_1{x}_i\right)}{1+\mathit{\exp}\left({a}_0+{a}_1{x}_i\right)}$, where *a*_1_ ∈ *ℝ*^+^ to ensure that the probability of DLT monotonically increases with dose levels. For additional information on parameter estimation, please see the article by Babb et al. (1998), which includes a re-parametrization of this approach in terms of MTD and the probability of DLT at the initial dose level.

### Time-to-event EWOC (TITE-EWOC)

TITE-EWOC is a hybrid design that introduces the time-to-event approach in the EWOC method. The aim of this design is to enable continuous recruitment and to decrease the dose-finding trial duration without impairing the characteristics of the EWOC design, especially its ability to control against overdose.

### GUIP1 implementation

The different dose escalation methods discussed above are implemented in different libraries in the R software. The dfcrm library allows the CRM method to be applied with the crm function. The choice of CRMB or CRML is made using the “method” parameter. Two dose-toxicity models are proposed: a one-parameter logistic model with fixed intercept and an empirical model. The titecrm function of this library implements the TITE-CRM [7]. The EWOC method [8] can be applied with the bcrm function of the bcrm library, which allows the dose-toxicity relationship to be modeled with several functional forms. We implemented the TITE-EWOC method and integrated it into GUIP1, as it was only available upon request to the authors.

**Fig. 1:**
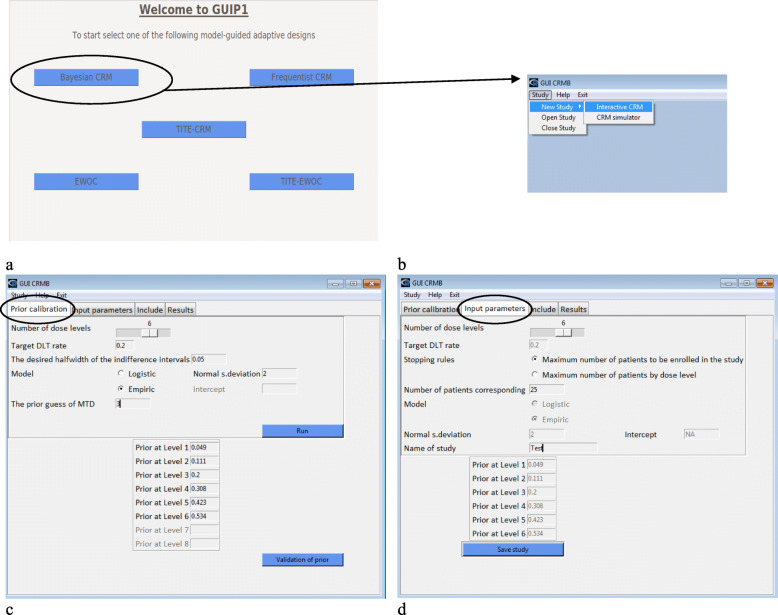
Implementation of GUIP1 / CRMB (motivating example)

GUIP1 was built using the TCL/TK programming language, which can be compiled via the R software libraries tcltk, tkrplot, and tcltk2. It implements the methods previously described using five main functions: CRML, CRMB, TITE-CRM, EWOC, and TITE-EWOC. Using the GUIP1 package, the interface can be launched using the command GUIP1() (Fig. 1a, b). A long form of the documentation (vignette) was also developed to facilitate the use of GUIP1.

## Results

### Motivating example

A motivating example is used to describe the program. This section describes the required clinical inputs for implementing CRMB (Fig. 1c, d) as well as CRML, TITE-CRM, EWOC, or TITE-EWOC (supplementary Fig. 1a, b, c, d, respectively). Six dose levels {*x*_1_, …, *x*_6_} were chosen, where *x*_3_ was the assumed dose level associated with the target toxicity level of 20% (i.e., *θ* = 0.2). The prior estimates of the probability of patients who would experience a DLT, at each dose level from 1 to 6, were 0.049, 0.111, 0.2, 0.308, 0.423, and 0.534, respectively. It was assumed that patients were followed over 12 weeks for time-to-event designs and over 4 weeks for the other designs. A one-parameter model (empiric or logistic) was chosen in both cases. For the EWOC method, the probability of exceeding the target dose α was set to 0.25. The stopping rule of the study corresponded to the maximum number of patients who could be enrolled in the study and was fixed at 25 (Fig. 1c, d).

### The CRMB and CRML functions

**Fig. 2:**
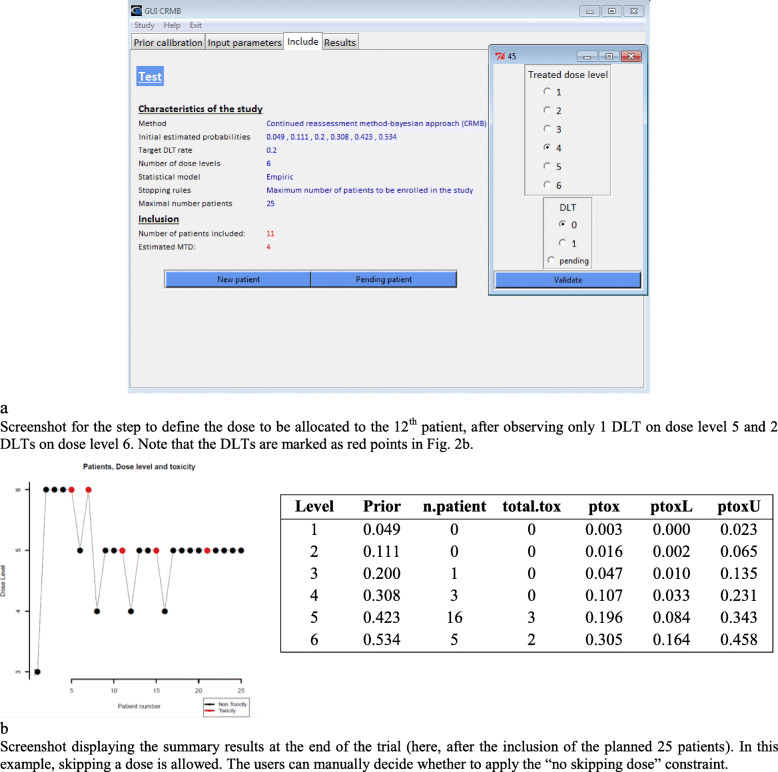
Conducting a study with GUIP1 / CRMB (motivating example)

The views obtained for both the CRMB and CRML functions are similar. The “Interactive CRM” tab allows a new study to be started or an ongoing study to be opened (Fig. 1b). Using the “Prior calibration” tab (Fig. 1c), the following different clinical input parameters are defined: number of dose levels, target toxicity level, desired halfwidth of the indifference intervals, dose-toxicity model, parameters related to the chosen model, and the starting dose level (assumed MTD level). The acceptable toxicity probabilities associated with each dose level were estimated by clicking on the “Run” button using the getprior function (package dfcrm) according to the model calibration approach [23]. The acceptable toxicity probabilities can be changed manually by the users. Once the toxicity probabilities are validated, we complete the additional trial parameters: the stopping rule and its related number of patients which will define the maximum cohort size, as well as the study name (“Input parameters” tab, Fig. 1d). Two stopping rules are currently implemented. The first rule stops the inclusions if the maximum sample size is reached, and the second rule causes a stop if a predefined number of patients has been treated to a certain dose level. On the same tab, the “Save study” button generates two files in the working directory: “study_name.crmb” or “study_name.crml” contains different characteristics of the study, and “study_name-crml.Rdata” or “study_name-crmb.Rdata” contains the updated data after each patient assessment. Note that the extensions (.crmb or .crml) differ according to the function. In the “Include” tab (Fig. 2a), the “New patient” button allows patients to be enrolled in the trial. At each new inclusion, the user must provide the given dose level and the observed DLT response (0 = no and 1 = yes) or indicate “pending” if appropriate. The default dose level corresponds to the estimated dose closest to the MTD, taking into account the data from previous patients. The “Pending patient” button allows the patient data to be updated (DLT yes/no) once the response is known. After the first patient is included, the study parameters can no longer be modified. A summary of the results of the inclusions can be viewed at any time via the “Results” tab. Two tables summarizing the results per patient and dose level and a graph showing the dose escalation are displayed (Fig. 2b). These different results can be exported via the “Export” button, which creates an .xlsx file in the current directory that contains these two tables as well as the graphic in png format.

Our motivating example treated up to 25 patients. The starting dose was defined as dose level 3 and was given to the first enrolled patient. The CRMB ran sequentially for each patient. Each new patient was treated at the proposed dose level (closest to the MTD) and, at the end of the observation period, the occurrence (or not) of DLT was recorded. This step was repeated until the study stopped. Results are shown in Fig. 2b and summarized by patient and dose level.

### TITE-CRM

The operation of the function TITE-CRM is similar to that of the CRMB and CRML functions except for the “Input parameters” and “Include” tabs. Indeed, since this method accounts for the time until DLT observation, it is necessary to define the maximum observation period for all patients. This parameter is indicated in the “Observation Window” field of the “Input parameters” tab. Consequently, for each new inclusion, a variable recording the follow-up was added. An update of the follow-up is performed for all patients with incomplete observations. The default value is equal to the maximum observation period. Note that the value of patient follow-up must be smaller than the duration of the observation period. Supplementary Fig. 1a shows the view obtained for the TITE-CRM method.

### EWOC

Similar to the CRMB, CRML, and TITE-CRM methods, the parameters for the EWOC function are defined in the “Input parameters” tab. However, running this method using the bcrm function (package bcrm) [20] requires additional variables. This function allows the prior distribution of the model parameter to be selected as either a gamma, uniform, or lognormal distribution along with its respective associated parameters. Two supplementary parameters are added according to the EWOC method. The “pointest” parameter, between 0 and 0.5, provides overdose control and represents the maximum acceptable proportion of overdosed patients. A parameter “Dose skipping constraint” was also added to avoid skipping doses in escalation immediately after a toxic outcome. The “Include” and “Results” tabs are similar to those for the previous methods. Supplementary Fig. 1b shows the view obtained for the EWOC method.

### TITE-EWOC

The TITE-EWOC and EWOC views are quite similar. However, for TITE-CRM, in addition to basic input parameters, the “Observation window” is required to record the follow-up. The user can also enter an initial value for the parameter model with the field “Prior alpha value”. Given that the TITE-EWOC method accounts for the follow-up of each patient and some updated follow-ups are expected at the inclusion of new patients, we proposed some new functionalities for the “Include” tab. A “Modif patient data” button was added to update, if needed, the data of the previous patient included. The possibility of entering multiple patient data at the same time is now available by clicking on “Yes” in the pop-up display after selecting “New patient”. The data to be entered are “dose level,” “DLT,” and “Follow-up” of each new patient as comma-separated values. Note that it is always possible to enter patient data sequentially by clicking “No” in the pop-up display. Supplementary Fig. 1c shows the view obtained for the TITE-EWOC method.

### Simulating a study

**Fig. 3:**
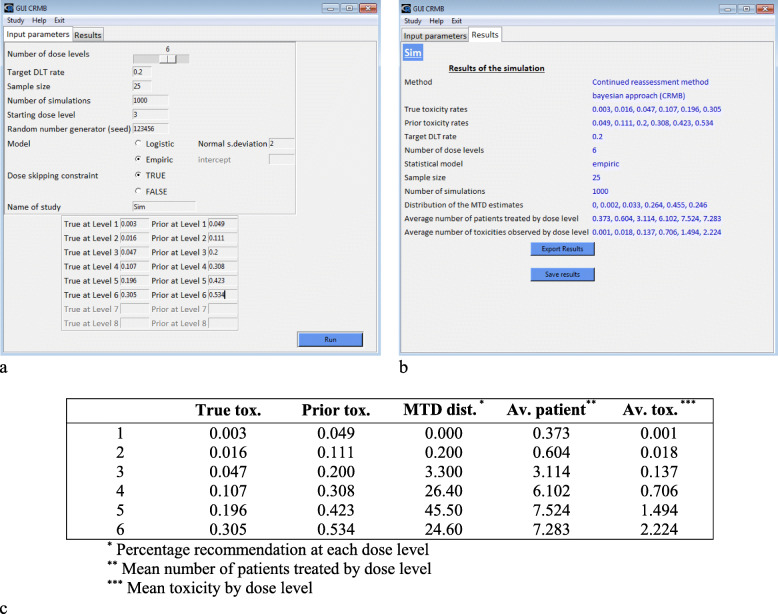
Simulation with GUIP1 / CRMB (motivating example)

We simulated 1000 trials of *n* = 25 patients according to the parameters used in the motivating example (Fig. 3). In addition, the probabilities of toxicity that would be observed in a real-life setting for each dose level were assumed to be the following: 0.003, 0.016, 0.047, 0.107, 0.196, and 0.305. In practice, it is usually recommended that several scenarios be considered. Dose level 5 (0.196) is the closest to the MTD (0.2).

The simulations revealed that the expected dose (dose level 3) is recommended (Fig. 3c, column “MTD dist.”) as the MTD (target DLT rate = 20%) in 3.3% of the experiments. Similarly, the dose levels 4, 5, and 6 are recommended in 26.4, 45.5, and 24.6% of the experiments, respectively. Dose levels 1 and 2 were shown to be poor or not recommended. In accordance with the true probabilities of toxicity and despite the wrong guess for the prior MTD, dose level 5 was the most recommended (45.5%). The number of patients treated at each dose level (Fig. 3c, column “Av. patient”) was distributed as follows: 0.373 patients (1.49%) at dose level 1, and 0.604 (2.42%), 3.114 (12.45%), 6.102 (24.41%), 7.524 (30.1%), and 7.283 (29.13%) at dose levels 2, 3, 4, 5, and 6, respectively. Four out of ten patients were treated at doses lower than the MTD, three out of ten at the MTD, and three out of ten at dose level 6 (overdose). The average results might be improved with better choices of input parameters or with another dose-escalation design. For instance, the overdose rate can be decreased using an EWOC design.

## Discussion

Several model-based dose-finding methods exist in oncology drug development. Despite a gradual increase in their use, these methods remain underutilized in practice due to their complexity.

Unlike the software already available for implementing dose-escalation designs for a phase I clinical trial, we developed an interface, GUIP1, available on GitHub (https://github.com/ddinart/GUIP1), that facilitates the use of these adaptive mathematical methods based on the modeling of the dose-toxicity relationship. GUIP1 is implemented using the free software R, which is widely used by statisticians in oncology. GUIP1 simplifies the use of these dose escalation methods and is designed to be fairly simple for beginners in R. Furthermore, its user-friendly interface is an easy-to-use solution that offers multiple possibilities.

GUIP1 provides the possibility of managing real clinical trials using file management options with automatic backup of study and/or simulation results. It uses libraries already published, tested, and validated by the scientific community and allows access to the data at all times for further analysis if necessary. In addition to being an interface for running statistical dose-escalation models, GUIP1 allows the real-time management of phase I clinical trials with an automatic backup of the results and the ability to export .xlsx files. The export module returns the results of a study or simulations. This service provides full traceability of the study and is very convenient. The tools used to develop GUIP1 are very flexible, and this makes it possible to update the software at any time by adding new methods not yet implemented under R or by adding stopping criteria recently published in the literature. The stopping rules implemented in our interface do not extend beyond the existing rules in the package bcrm. Other stopping rules, including those based on binary trees, have been suggested [24]; however, they have not been made available in the interface. Therefore, adding more existing rules would be an interesting enhancement.

## Conclusions

One challenge with the conception of GUIP1 is a lack of information on the use of the tcltck package. Hence, developing the attractiveness and appeal of the interface as well as adding new functionality might be a future goal. We hope that this interface will help spread the adoption of new methods and increase their use in future therapeutic trials.

### Availability and requirements

**Project name:** GUIP1 Package

**Project home page:** https://github.com/ddinart/GUIP1

**Operating system(s):** Platform independent

**Programming language:** R

**Other requirements:** R v3.5.2

**License:** GPL (> = 2)

**Any restrictions to use by non-academics:** None

## Supplementary information

**Additional file 1: Figure 1.** Implementation of GUIP1 (a) CRML, (b) TITE-CRM, (c) EWOC, and (d) TITE-EWOC (motivating example).

## Data Availability

The interface GUIP1 is available on GitHub (https://github.com/ddinart/GUIP1).

## Abbreviations

BOIN: Bayesian optimal interval
CRM: Continual reassessment method
CRMB: Continual reassessment method with Bayesian estimation
CRML: Continual reassessment method with maximum likelihood estimation
DLT: Dose-limiting toxicity
EWOC: Escalation with overdose control
FIH: First-in-human
INCa: French National Cancer Institute
MTD: Maximum tolerated dose
mTPI: Modified toxicity probability interval
RP2D: Recommended phase II dose
TITE-CRM: Time-to-event-CRM
TITE-EWOC: Time-to-event EWOC
