# Supplementary material for: GUIP1: a R package for dose escalation strategies in phase I cancer clinical trials
Source: BMC Med Inform Decis Mak. 2020 Jun 24;20:134. doi: 10.1186/s12911-020-01149-3 (PMC7469913; doi:10.1186/s12911-020-01149-3)
Supplement: Supplementary file 1 — Additional file 1: Figure 1. Implementation of GUIP1 (a) CRML, (b) TITE-CRM, (c) EWOC, and (d) TITE-EWOC (motivating example). [file 12911_2020_1149_MOESM1_ESM.pdf]

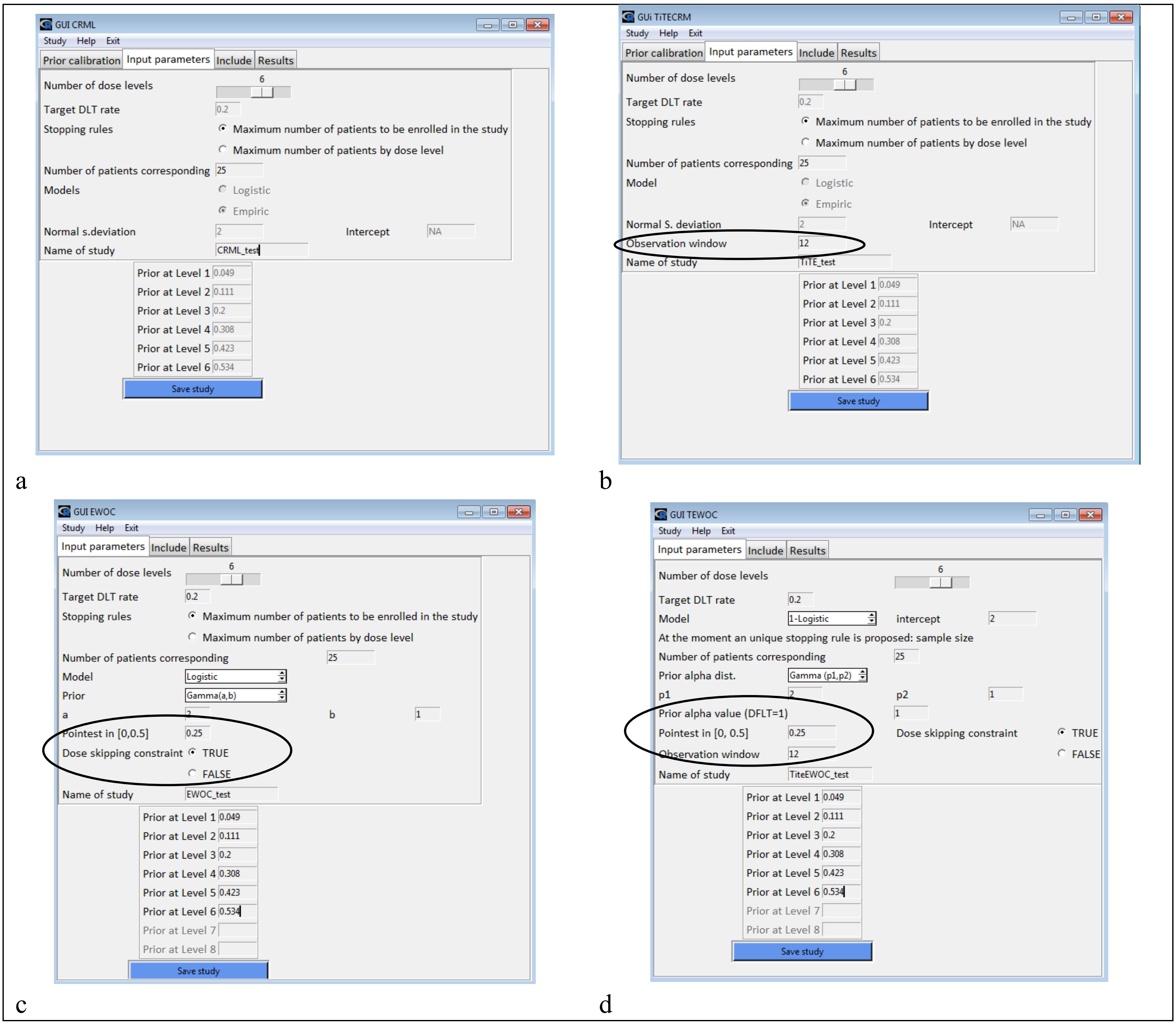
GUI CRML
Study Help Exit
Prior calibration
Input parameters
Include
Results
Number of dose levels
6
Target DLT rate
0.2
Stopping rules
Maximum number of patients to be enrolled in the study
Maximum number of patients by dose level
Number of patients corresponding
25
Models
Logistic
Empiric
Normal s.deviation
2
Intercept
NA
Name of study
CRML_test
Prior at Level 1 0.049
Prior at Level 2 0.111
Prior at Level 3 0.2
Prior at Level 4 0.308
Prior at Level 5 0.423
Prior at Level 6 0.534
Save study
a
GUi TITECRM
Study Help Exit
Prior calibration
Input parameters
Include
Results
Number of dose levels
6
Target DLT rate
0.2
Stopping rules
Maximum number of patients to be enrolled in the study
Maximum number of patients by dose level
Number of patients corresponding
25
Model
Logistic
Empiric
Normal S. deviation
2
Intercept
NA
Observation window
12
Name of study
TITE_test
Prior at Level 1 0.049
Prior at Level 2 0.111
Prior at Level 3 0.2
Prior at Level 4 0.308
Prior at Level 5 0.423
Prior at Level 6 0.534
Save study
b
GUI EWOC
Study Help Exit
Input parameters
Include
Results
Number of dose levels
6
Target DLT rate
0.2
Stopping rules
Maximum number of patients to be enrolled in the study
Maximum number of patients by dose level
Number of patients corresponding
25
Model
Logistic
Prior
Gamma(a,b)
a
2
b
1
Pointest in [0,0.5]
0.25
Dose skipping constraint
TRUE
FALSE
Name of study
EWOC_test
Prior at Level 1 0.049
Prior at Level 2 0.111
Prior at Level 3 0.2
Prior at Level 4 0.308
Prior at Level 5 0.423
Prior at Level 6 0.534
Prior at Level 7
Prior at Level 8
Save study
c
GUI TEWOC
Study Help Exit
Input parameters
Include
Results
Number of dose levels
6
Target DLT rate
0.2
Model
1-Logistic
intercept
2
At the moment an unique stopping rule is proposed: sample size
Number of patients corresponding
25
Prior alpha dist.
Gamma (p1,p2)
p1
2
p2
1
Prior alpha value (DFLT=1)
1
Pointest in [0, 0.5]
0.25
Dose skipping constraint
TRUE
FALSE
Observation window
12
Name of study
TiteEWOC_test
Prior at Level 1 0.049
Prior at Level 2 0.111
Prior at Level 3 0.2
Prior at Level 4 0.308
Prior at Level 5 0.423
Prior at Level 6 0.534
Prior at Level 7
Prior at Level 8
Save study
d
